# Forschung und Praxis des Kinder- und Jugendsports im Dialog

**DOI:** 10.1007/s43594-020-00004-7

**Published:** 2020-10-16

**Authors:** Jan Holze, Nils Neuber, Peter Lautenbach

**Affiliations:** 1 Deutsche Sportjugend, Schwerin, Deutschland; 2 Westfälische Wilhelms-Universität Münster, Münster, Deutschland; 3 Deutsche Sportjugend, Frankfurt am Main, Deutschland

Liebe Leserinnen und Leser,

wir freuen uns, Ihnen und euch die erste Ausgabe von *Forum Kinder- und Jugendsport – Zeitschrift für Forschung, Transfer und Praxisdialog *vorlegen zu können. Bewegung, Spiel und Sport gehören zu den häufigsten und wichtigsten Aktivitäten im Aufwachsen von Kindern und Jugendlichen. Dennoch gibt es im deutschsprachigen Raum zwar einige Periodika zum Schulsport, aber keine Zeitschrift, die sich explizit mit dem Kinder- und Jugendsport befasst. Hier setzt das *Forum Kinder- und Jugendsport – Zeitschrift für Forschung, Transfer und Praxisdialog* an. Unsere neue Publikation fördert den systematischen Forschungs-Praxis-Dialog, indem sowohl Forschungsbeiträge mit wissenschaftlichem Qualitätsstandard als auch Fachbeiträge aus der Praxis des Kinder- und Jugendsports und der allgemeinen Kinder- und Jugendarbeit veröffentlicht werden.

Die Forschungsbeiträge werden von unabhängigen Herausgeber*innen aus dem Forschungsverbund der Deutschen Sportjugend betreut und durchlaufen ein double-blind Peer-Review-Verfahren, wie es bei wissenschaftlichen Zeitschriften üblich ist. Im Heft werden sie aus jeweils unterschiedlicher Perspektive kommentiert und eingeordnet, um vielfältige Bezüge zwischen Forschung und Praxis herzustellen. Die Fachbeiträge und ergänzenden Rubriken werden von einer Redaktion der Deutschen Sportjugend koordiniert. Die Deutsche Sportjugend (dsj) ist der größte freie Träger der Kinder- und Jugendhilfe in der Bundesrepublik Deutschland, sie führt das jugendpolitische Mandat des Deutschen Olympischen Sportbunds und entwickelt unter aktiver Mitbestimmung junger Menschen zeitgemäße Rahmenbedingungen für den Kinder- und Jugendsport.

Mit seinem innovativen interdisziplinären Ansatz richtet sich das *Forum Kinder- und Jugendsport *an ehrenamtliche und hauptberufliche Mitarbeiter*innen des Kinder- und Jugendsports, an Wissenschaftler*innen mit Schwerpunkt im Kinder- und Jugendsport und in der allgemeinen Jugendforschung, an Student*innen sowie an Sport‑, Bildungs- und Jugendpolitiker*innen. Wir hoffen, mit diesem neuen Format Beiträge zur Stärkung und Weiterentwicklung des Kinder- und Jugendsports zu leisten – indem wir die bestehende Praxis beschreiben und evaluieren, hinterfragen und kritisieren, bestätigen und innovieren. Denn nur wer hin und wieder prüft, erlangt auf lange Sicht Handlungssicherheit.

Das vorliegende erste Heft enthält drei Forschungsbeiträge. *Tim Bindel *und *Christian Theis* befassen sich in einer ethnografischen (Selbst‑)Studie mit den bestimmenden Merkmalen der aktuellen Fitnesskultur junger Menschen. Als langjähriger Kenner und Protagonist bilanziert *Wolf-Dietrich Brettschneider *zentrale Entwicklungen aus 30 Jahren sportvereinsbezogener Kinder- und Jugendforschung und wagt einen Ausblick auf die Zukunft. *Dennis Dreiskämper, Till Utesch, Lena Henning, Nina Ferrari, Christine Graf, Maike Tietjens und Roland Naul *stellen eine aktuelle Studie zum Zusammenhang von motorischer Leistungsfähigkeit, körperbezogenem Selbstkonzept und Body-Mass-Index in Kindergarten und Grundschule vor.

Ergänzt werden die Forschungsbeiträge durch eine Stellungnahme aus dem gemeinnützigen, organisierten Sport (*Peter Lautenbach*) zum Jugendsporttrend Fitness, durch ein Interview mit dem Erziehungswissenschaftler *Ivo Züchner* zum Stand der Jugendforschung und der Rolle des Sports dabei sowie durch Fragen an den Vorstandsvorsitzenden des Niedersächsischen Instituts für frühkindliche Bildung und Entwicklung, *Jan Erhorn*. Abgerundet wird die Premiere von *Forum Kinder- und Jugendsport* mit Fachbeiträgen des Reinickendorfer Bezirksstadtrats *Tobias Dollase*, Vorstandsmitglied der dsj, zur Förderung von Bewegung und Sport in der Kommune, von *Ulrike Hoffmann* zur Nachhaltigkeit im Sportverein, von *Rüdiger Bockhorst* und *Anika Krumhöfner* zur Förderung der Ganztags-Kooperation zwischen Sportverein und Schule im Landkreis Gütersloh sowie durch unser „Aktuelles Stichwort“. In dieser Rubrik erörtert *Katharina Morlang* den Megatrend Digitalisierung und wie Jugendsportorganisationen den digitalen Wandel angehen.

Das Erscheinen des ersten Hefts von *Forum Kinder- und Jugendsport* hat sich um einige Monate verzögert. Zum Zeitpunkt der Planung der Beiträge stand die Covid-19-Pandemie noch nicht auf der Weltagenda. Aus diesem Grund finden sich in dieser Ausgabe noch keine Betrachtungen mit Bezug zum Coronavirus und dessen Auswirkungen auf den Kinder- und Jugendsport. Im zweiten Heft werden wir diese Aufgabe angehen.

Wir hoffen, mit dem ersten Heft ein breites Themenspektrum des Kinder- und Jugendsports abzubilden und wünschen Ihnen und euch eine angenehme und bereichernde Lektüre.

Jan Holze (für die Institutionellen Herausgeber*innen)

Nils Neuber (für die Herausgeber*innen der Forschungsbeiträge)

Peter Lautenbach (für die Herausgeber*innen der Fachbeiträge)

